# Maintenance of Synaptic Stability Requires Calcium-Independent Phospholipase A_2_ Activity

**DOI:** 10.1155/2012/569149

**Published:** 2012-05-20

**Authors:** Julie Allyson, Xiaoning Bi, Michel Baudry, Guy Massicotte

**Affiliations:** ^1^Département de Chimie-Biologie, Université du Québec à Trois-Rivières, 3351 Boulevard des Forges, QC, Canada G9A 5H7; ^2^Graduate College of Biomedical Sciences, Western University of Health Sciences, Pomona, CA 91766-1854, USA

## Abstract

Phospholipases A_2_ (PLA_2_s) represent one of the largest groups of lipid-modifying enzymes. Over the years, significant advances have been made in understanding their potential physiological and pathological functions. Depending on their calcium requirement for activation, PLA_2_s are classified into calcium dependent and independent. This paper mainly focuses on brain calcium-independent PLA_2_ (iPLA_2_) and on the mechanisms by which they influence neuronal function and regulate synaptic plasticity. Particular attention will be given to the iPLA_2_ **γ** isoform and its role in the regulation of synaptic glutamate receptors. In particular, the paper discusses the possibility that brain iPLA_2_ **γ** deficiencies could destabilise normal synaptic operation and might contribute to the aetiology of some brain disorders. In this line, the paper presents new data indicating that iPLA_2_ **γ** deficiencies accentuate AMPA receptor destabilization and tau phosphorylation, which suggests that this iPLA_2_ isoform should be considered as a potential target for the treatment of Tau-related disorders.

## 1. Introduction

The nervous system is formed by integrated neuronal circuits which all require constant adaptation for stabilizing their activities in the face of perturbations that alter, for instance, neuronal excitability. Phenomena that conform to this definition include the activity-dependent regulation of intrinsic neuronal firing properties [1, 2], pre- and postsynaptic forms of excitatory synaptic plasticity, such as synaptic scaling, that adjust all of a neuron's excitatory synapses up or down in the right direction to stabilize firing [3, 4]; the balancing of excitation and inhibition within neuronal networks [5, 6]; compensatory changes in synapse number [7]; apposition of presynaptic and postsynaptic elements [4] and metaplastic mechanisms that adjust long-term changes in synaptic operation [8, 9]. In general, it is believed that the final refinements of neuronal circuits rely on the stabilization of functionally appropriate connections and the elimination of inappropriate ones.

While the molecular mechanisms of synapse formation have been extensively studied, very little is known about the molecular mechanisms that are responsible for stabilization of synaptic connections. Over the recent years, however, it has been proposed that the level of AMPA subtype of glutamate receptors found at neuronal connections might be a crucial component controlling both stabilization of presynaptic inputs and postsynaptic spine morphogenesis (see [10]). In the present paper, we will focus on the possibility that a specific PLA_2_ isoform can interact with AMPA receptor properties to contribute to synaptic stabilization. We will, in this line, present some new information indicating that iPLA_2_ *γ* deficiency might undermine the normal stabilizing mechanisms underlying memory formation in the hippocampus and contribute to Alzheimer's disease pathology.

## 2. iPLA_2_ Isoforms, Long-Term Potentiation, and Memory Stabilization

Phospholipases A_2_ (PLA_2_s) constitute a large and diverse group of enzymes with broad biological functions, ranging from membrane synthesis and turnover to the generation of signaling molecules. So far, more than 20 isoforms of PLA_2_ with diverse characteristics, including calcium requirement and subcellular localization, have been identified. Based on nucleotide sequences as well as other properties, PLA_2_s have been categorized into 15 groups (I–XV) [11, 12]. Several types of released small PLA_2_s (~14 kDa) require millimolar amounts of calcium for optimal activation. These enzymes have historically been called the secreted forms of PLA_2_ (or sPLA_2_). The remaining groups are larger proteins, localized in intracellular compartments, and are either calcium dependent or independent.

The first intracellular PLA_2_ to be cloned was a protein of 85-kDa, classified as group IV PLA_2_ [13, 14]. This enzyme, now designated as cytosolic PLA_2_ *α* (cPLA_2_ *α*), is under the influence of extracellular signals likely to induce calcium mobilization and phosphorylation reactions [13]. Another group of PLA_2_ (group VI), which does not require calcium variations for activity, has been cloned [15–17]. This PLA_2_ isoform has been designated as calcium-independent PLA_2_ (iPLA_2_) (Table 1) [14, 18], and according to numerous lines of biochemical evidence may account for most PLA_2_ activity under resting conditions. Whereas cPLA_2_ and sPLA_2_ are commonly believed to be preferentially involved in AA release, emerging evidence indicates that iPLA_2_ activity can contribute to docosahexaenoic acid (DHA) release from brain phospholipids. Pharmacologically, iPLA_2_ activity is markedly reduced by bromoenol lactone (BEL), a suicide substrate, which is not an effective inhibitor of sPLA_2_ or cPLA_2_ at comparable concentrations [15, 19]. Several interesting reviews have considered the physiological and pathological functions of PLA_2_ enzymes [19–30]. In this paper, we describe new and unique functional roles of iPLA_2_ in the regulation of brain glutamate receptor functions, neuronal plasticity, and neurodegenerative processes.

Among PLA_2_ enzymes, group IV (cPLA_2_) and group VI (iPLA_2_) families represent intracellular enzymes with a catalytic serine in their lipase consensus motif. Various studies, including gene targeting, have indicated that group IV_A_ cPLA_2_ (cPLA2*α*), which is regulated by calcium-dependent membrane translocation and mitogen-activated protein kinase- (MAPK-) dependent phosphorylation, is essential for stimulus-dependent eicosanoid biosynthesis [31, 32]. On the other hand, group VI_A_ iPLA_2_ (iPLA2*β*) and group VI_B_ iPLA_2_ (iPLA_2_ *γ*) isoforms mainly exhibit phospholipase activity, whereas the other iPLA_2_ isoforms *δ*, *ε*, *ξ*, and *η* display triglyceride lipase and transacylase activities (see Table 1) [33, 34]. Members of this family share a protein domain discovered initially in patatin, the most abundant protein of the potato tuber. Patatin (also called iPLA2*α*) is a lipid hydrolase with an unusual folding topology that differs from classical lipases. The catalytic dyad (Ser-Asp) rather than the catalytic triad (Ser-His-Asp) is found in classical lipases and does not contain a lid domain usually required for interfacial activation [35]. The catalytic dyad is located in a conserved catalytic domain that shows 40% homology with patatin/iPLA2*α*. Adjacent to the catalytic center, there is a conserved nucleotide binding motif. The iPLA-type enzymes (i.e., iPLA2*β* and iPLA2*γ*) typically possess a long N-terminal domain, which may be involved in protein-protein interaction, distinct translation, and membrane spanning. Like numerous proteins containing the uptake-targeting Ser-Lys-Leu (SKL) sequence, it has been found that iPLA2g tightly associated with peroxisomal membranes [36]. The lipase-type enzymes (i.e., iPLA_2_ isoforms *δ*, *ε*, *ξ* and *η*) lack the N-terminal domain and are thought to act primarily on triglycerides or other neutral lipids in lipid droplets [37]. Very little is known about the developmental brain expression of iPLA_2_ in the brain. In mouse, expression of iPLA_2_ enzymes has been reported in sagittal sections at embryonic day 14.5 [38]. At this stage, the strongest expression seen in the brain is in the alar plate of the developing hindbrain with prominent expression also in an analogous region of the midbrain. IPLA_2_s also appear to be expressed in the developing diencephalon and telencephalon of the forebrain. In situ hybridization studies have revealed that across several stages of human embryonic and early fetal development, iPLA_2_s show a dynamic expression pattern both in terms of the location of expression and the differentiation state of expressing cells. In brief, iPLA_2_s are expressed in forebrain and midbrain before it is detectable in hindbrain. Throughout the developing brain, iPLA_2_s are mainly expressed in proliferative zones, suggesting that these enzymes are important for early neuronal development [38]. The precise pattern of expression of both group VI_A_ iPLA_2_ (iPLA2*β*) and group VI_B_ iPLA_2_ (iPLA_2_ *γ*) enzymes still unclear, and one important priority for future studies will be the precise identification of iPLA_2_ isoforms responsible for brain development and stabilization.

Group VI_A_ iPLA_2_ *β*, the most extensively studied iPLA_2_ isoform, has been implicated in various cellular events, such as phospholipid remodelling [18, 39], eicosanoid formation [40], cell growth [41, 42], apoptosis [43], and activation of store-operated channels and capacitative calcium influx [44]. Disruption of the iPLA_2_ *β* gene causes impaired sperm motility [45], mitigated insulin secretion [46, 47], and neuronal disorders with iron dyshomeostasis [48]. Group VI_B_ iPLA_2_ *γ* is a membrane-bound iPLA_2_ enzyme with unique features, such as the utilization of distinct translation initiation sites producing different sizes of enzymes with distinct subcellular localizations [36, 49–53] and phospholipid selectivity in terms of sn-1/sn-2 positional specificity, which differs among substrates [54]. iPLA_2_ *γ* has a mitochondrial localization signal in the N-terminal region and a peroxisomal localization signal near the C-terminus, and the 88-kDa full-length and 63-kDa translation products of iPLA_2_ *γ* are preferentially distributed in mitochondria and peroxisomes, respectively [49–51]. In brain, iPLA_2_ constitutes the predominant phospholipase activity under resting conditions [55, 56]. Reverse transcription-polymerase chain reaction experiments have revealed that rat brain constitutively expresses mRNAs for at least 3 calcium-independent PLA_2_ isoforms, iPLA_2_ *β*, iPLA_2_ *γ* and cPLA_2_ *γ* [16, 57, 58]. These isoforms are characterized by different sensitivity to PLA_2_ inhibitors, including different enantiomers of an inhibitor; Jenkins et al. [59] established that the (S)-enantiomer of BEL selectively reduces iPLA_2_ *β* activity, while its (R)-enantiomer blocks the iPLA_2_ *γ* isoform more efficiently.

Although little is known about iPLA_2_ functions in neurons, a growing body of evidence suggests their involvement in hippocampal long-term potentiation (LTP) of excitatory synaptic transmission [55, 60]. Hippocampal LTP, first described by Bliss and Lomo in 1973, is commonly regarded as a functional model of synaptic adaptation (i.e., plasticity) that likely participates in certain forms of learning and memory [61–63]. PLA_2_ activities are increased in membranes prepared from the dentate gyrus after LTP induction in anaesthetized rats [64]; it has been proposed that PLA_2_ could be involved in hippocampal LTP expression by elevating the production of arachidonic acid (AA) that could retrogradely increase transmitter release at glutamatergic synapses [65, 66]. The hypothesis that facilitation of transmitter release by PLA_2_s occurs during LTP is reinforced by the fact that iPLA_2_ activity plays an important role in membrane fusion processes required for exocytosis [21, 67].

The notion that iPLA_2_ activity may facilitate LTP expression by increasing glutamate release is contradicted, however, by a number of reports demonstrating that synaptic potentiation, at least in area CA1 of hippocampus, is not dependent on changes in transmitter release, but rather is mediated by upregulation of postsynaptic responses mediated by alpha-amino-3-hydroxy-5-methyl-4-isoxazole-propionic acid (AMPA) receptors at glutamatergic synapses [68, 69]. Several alterations have been reported at postsynaptic sites during LTP, including faster kinetics of receptor ion channels [70, 71], redistribution of existing receptors within the postsynaptic density [72], and insertion of new receptors at synapses [73, 74]. Consistent with these observations, we recently demonstrated that pretreatment of hippocampal slices with the iPLA_2_ inhibitor BEL completely abolishes AMPA receptor translocation in synaptic membranes and expression of CA1 hippocampal LTP [75]. Interestingly, both LTP and AMPA receptor translocation display enantio-selective impairment by the iPLA_2_ *γ* blocker (R)-BEL but not by the iPLA_2_ *β* inhibitor (S)-BEL, suggesting that iPLA_2_ *γ* represents the crucial isoform governing hippocampal synaptic stability. iPLA_2_ *γ* mRNAs and proteins are enriched with the endoplasmic reticulum (ER)-Golgi apparatus in several cell types [57], where they may be essential for diverse intracellular trafficking pathways, such as retrograde movement from the Golgi complex to the ER, transport of material from the trans-Golgi network to the plasma membrane, or recycling of membranes and receptors through endocytic pathways [21]. In particular, Péchoux et al. [76] reported that iPLA_2_ inhibition slowed down the transport of caseins from the ER to the Golgi apparatus and from the trans-Golgi network to the plasma membrane, indicating that iPLA_2_ could participate in membrane trafficking events leading to the secretion of milk proteins. Since AMPA receptors trafficking from the ER-Golgi complex to postsynaptic membranes is critically involved in LTP [77], the iPLA_2_ *γ* isoform may be well suited to facilitate AMPA receptor translocation from intracellular pools to synaptic membranes during LTP.

Animal experiments have revealed that PLA_2_ inhibition resulted in synaptic plasticity impairment and decreased performance in memory tasks. For instance, intracerebral injection of wide-spectrum PLA_2_ inhibitors into chick intermediate medial hyperstriatum ventrale curbs the learning of a passive avoidance task [78], while intraperitoneal injection in rats impedes spatial learning in the Morris water maze [79]. Likewise, intracerebroventricular or intrahippocampal injection of specific iPLA_2_ inhibitors impairs spatial working memory in rodents [80]. In addition, acquisition of 1-trial step-down inhibitory avoidance in rats was shown to be correlated with increased iPLA_2_ activity in hippocampus, while bilateral injection of iPLA_2_ inhibitors in region CA1 of the dorsal hippocampus prior to training hindered both short-term and long-term memory [81]. In a modified protocol developed to test memory retrieval, the same group recently showed that injection of the dual cPLA_2_ and iPLA_2_ inhibitor palmitoyl trifluoromethylketone in region CA1 of the rat dorsal hippocampus before performance testing impaired trained behaviour in the step-down inhibitory avoidance task [82]. Importantly, memory retrieval was re-established after recovery of PLA_2_ activity, indicating that these PLA_2_s are indeed necessary for memory stabilization. Hence, intact iPLA_2_ activity seems to be critical for proper memory acquisition as well as retrieval. However, the identity of iPLA_2_ isoforms involved in memory acquisition and retrieval remains to be determined.

## 3. iPLA_2_ and Neuronal Cell Death Mechanisms

Recently, evidence from studies with nonneuronal cells has suggested that iPLA_2_ enzymes may have diverse effects on cell death. First, constitutive iPLA_2_ activity may contribute to cell death since iPLA_2_ *β* overexpression amplifies thapsigargin-induced apoptosis in INS-1 insulinoma cells [83] and accelerates U937 cell death after long-term exposure to hydrogen peroxide [84]. iPLA_2_ has been shown to play a pivotal role in oxidative damage of astrocytes [85], and its blockade by BEL dampens oligomeric amyloid-*β*- (A*β*1-42-) induced mitochondrial membrane potential loss and reactive oxygen species production in these cells [86]. Moreover, iPLA_2_ inhibition reduces the size of infarcts produced by global ischemia [87]. On the other hand, iPLA_2_ activity has also been shown to protect against cell death, as inhibition of iPLA_2_ accentuates oxidant-induced cell death in renal proximal tubule cells and astrocytes [88, 89]. Likewise, iPLA_2_ activity may also have deleterious or beneficial effects on neurons. For instance, acute inhibition of iPLA_2_ activity by racemic BEL has been found to be neuroprotective in organotypic hippocampal slices exposed to oxygen-glucose deprivation [90]. In contrast, immature cultures of primary cortical neurons exposed for several days to BEL showed decreased cellular viability and neuritic growth [91, 92]. Moreover, iPLA_2_ *β* knockout mice exhibit abnormal motor behaviors possibly related to the appearance of vacuoles and ubiquitin-positive axonal swelling (spheroids) in many brain regions [93, 94], suggesting that iPLA_2_ *β* dysfunction leads to axonal dystrophy.

While the reported impact of iPLA_2_ on cell viability is mostly attributable to iPLA_2_ *β*, involvement of the iPLA_2_ *γ* isoform is much less clear. A previous report demonstrated that iPLA_2_ *γ* localized in mitochondria catalyzed AA liberation that mediated mitochondrial permeability transition, a key control point for apoptosis [95]. On the other hand, iPLA_2_ *γ* expression may exert cytoprotective effects during complement-mediated glomerular epithelial cell injury [96]. In addition, recent findings from our laboratory have revealed that constitutive iPLA_2_ *γ* activity might represent an important neuroprotective system capable of limiting brain excitotoxic damage. In particular, we showed that iPLA_2_ *γ* inhibition by the enantio-specific inhibitor (R)-BEL makes cultured hippocampal slices more vulnerable to AMPA-mediated excitotoxicity [97]. Overactivation of N-methyl-D-aspartic acid (NMDA) or AMPA receptors results in a massive entry of calcium into cells, leading to the activation of a number of enzymes, including ATPases, lipases, proteases, and endonucleases that, in turn, deplete energy stores or damage cell membranes, cytoarchitecture or nuclear components, respectively. Excitotoxicity has been reported to contribute to a variety of neuropathological disorders, including ischemic stroke, epilepsy, amyotrophic lateral sclerosis, and Alzheimer's disease (AD) [98, 99].

Interestingly, iPLA_2_ *γ* inhibition-induced enhancement of AMPA-mediated toxicity is associated with selective phosphorylation and upregulation of the AMPA receptor GluR1 but not GluR2 subunits in synaptic membrane fractions [97, 98, 100]. In hippocampus, AMPA receptors generally form heterodimers containing 2 copies of each of the GluR1 and GluR2 subunits. It is now well-recognized that the presence of GluR2 subunits render AMPA receptors impermeable to calcium. Consequently, its presence or absence plays a critical role in cellular calcium homeostasis and in determining susceptibility to excitotoxicity [101, 102]. Hence, iPLA_2_ *γ* inhibition, by promoting surface expression of GluR1 over GluR2 subunits (which is reflected by a rise in the GluR1/GluR2 ratio in the membrane fraction), could exacerbate excitotoxic cell death through the increased formation of GluR2-lacking AMPA receptors that would allow adverse Ca^2+^ influx upon prolonged AMPA receptor activation. Consistent with this possibility, the greater cell death observed following iPLA_2_ *γ* inhibition is prevented by GluR1/3-specific AMPA receptor antagonists [97]. How inhibition of iPLA_2_ *γ* influences the expression of AMPA receptor subtypes in synaptic membranes remains an open question. As mentioned earlier, this may be the result of an effect of the lipase on protein transport through intracellular secretory pathways [76]. There are other circumstances in which GluR1 subunits are selectively upregulated in hippocampal neurons, such as after neuronal activity inhibition elicited by prolonged blockade of AMPA receptors [103] or by tumor necrosis factor-alpha receptor activation [104]. In the latter case, it has been proposed that upregulation of GluR1 homomeric receptors could be produced by a reserve pool of non-GluR2-containing AMPA receptors located near the membrane. Independently of the exact mechanism, these observations raise the possibility that constitutive iPLA_2_ *γ* activity may be a crucial mechanism to maintain synaptic stability and constitute a molecular device to prevent neuronal dysfunctions.

## 4. iPLA_2_ Dysfunction and Neurodegenerative Disorders

As previously described, cPLA_2_ and sPLA_2_ are commonly believed to be preferentially involved in AA release; emerging evidence indicates that iPLA_2_ activity can contribute to docosahexaenoic acid (DHA) release from brain phospholipids [105]. The first suggestion that brain iPLA_2_ activity may be crucial for DHA release came from a study by Strokin et al. [106] who showed that racemic BEL inhibited DHA release from astrocytes. Later, using siRNA silencing procedures, the same group demonstrated that DHA release from astrocytic phospholipids was mainly dependent on iPLA_2_ *γ* activity [107]. DHA is one of the most abundant omega-3 polyunsaturated fatty acids (PUFA) present in phospholipids of mammalian brain [108], where it is recognized to be important for the maintenance of neural membranes and brain function integrity [109]. Deficiency in dietary intake of DHA has been associated with lower performance in learning tasks in rodents [110–112]. On the other hand, DHA dietary supplementation was shown to decrease the risk of developing AD [113–115] and to exert neuroprotective actions in a mouse model presenting numerous aspects of Parkinson's disease [116], while high-fat consumption combined with low omega-3 PUFA intake promoted AD-like neuropathology [117]. Both iPLA_2_ activity and DHA levels have been reported to be decreased in the plasma of AD patients [118, 119]. Lower iPLA_2_ activity has also been reported in AD brains [120, 121]. Whether or not decreased iPLA_2_ *γ* activity is a factor contributing to AD pathology remains to be established. Numerous neurobiological studies have demonstrated that DHA may be acting in different cellular pathways to counteract several molecular manifestations of AD. There are, for instance, strong indications that DHA release in the brain may diminish oxidative stress [122, 123] and glutamate-induced toxicity [124]. In this line, DHA-induced reduction of excitotoxic damage in hippocampus might be dependent on internalization of AMPA receptors [125]. The potential ability of DHA to reduce caspase activation [114, 115], A*β* peptide accumulation, and Tau hyperphosphorylation [126, 127] also strongly supports the notion that DHA deficiency, as a result of iPLA_2_ deficiency, could represent a precursor event that could initiate the cellular manifestations of AD pathology.

**Figure 1:**
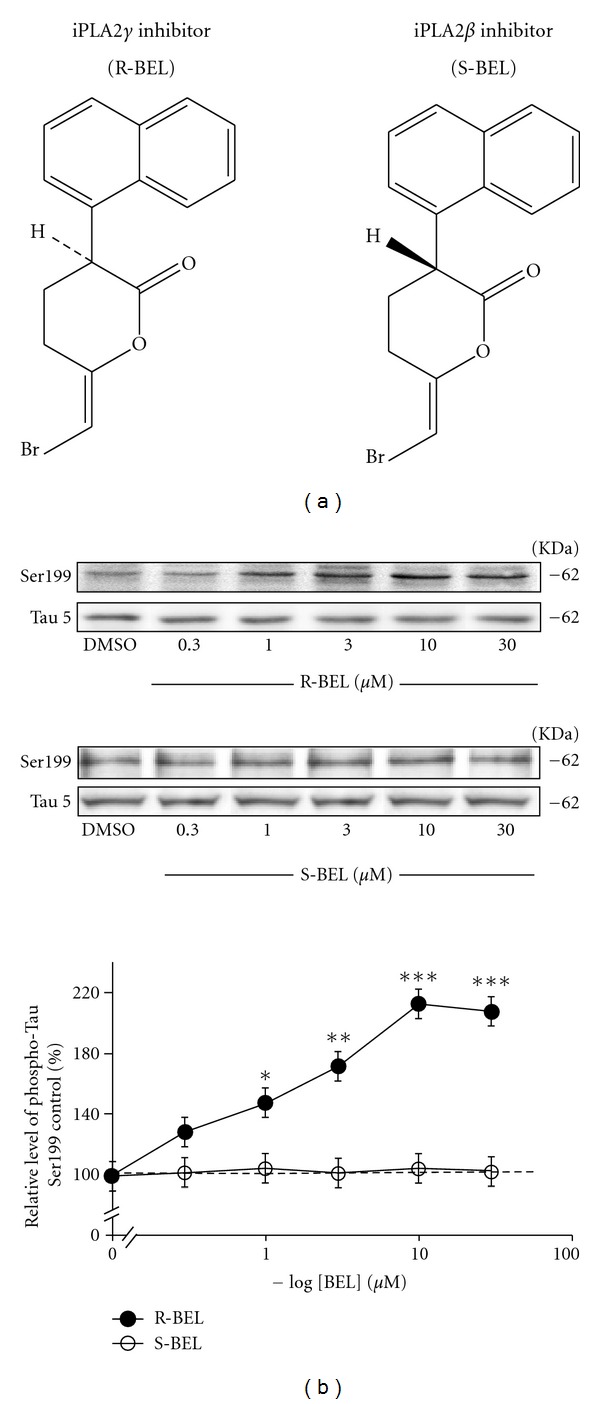
Hippocampal Tau phosphorylation at Ser199 residue is accentuated by R-BEL. Hippocampal slices (350 *μ*m) were pre-incubated at 32°C for 3 h with DMSO alone (control) or with increasing concentrations of the iPLA_2_ *γ* inhibitor R-BEL or the iPLA_2_ *β* inhibitor S-BEL (chemical structures of both compounds are presented in (a)). (b) Phosphorylation and Tau protein levels were determined by Western blotting of cell extracts (40 *μ*g of proteins) obtained from acute hippocampal slices. Phosphorylated Tau levels, expressed relative to total Tau (i.e., Tau-5; AbCam, Cambridge, MA, USA. Dilution 1 : 500), were measured using antibodies raised against Tau phosphorylated at Ser199 (AbCam, Cambridge, MA, USA. Dilution 1 : 1,000). The data were expressed as percentage of control values and are means ± SEM of 3 measurements per cell extract obtained from 7 different rats. Statistical analysis was performed by one-way ANOVA followed by Neuman-Keuls' post hoc test. *P < 0.05, **P < 0.01, ***P < 0.001, drug-treated versus control.

**Figure 2:**
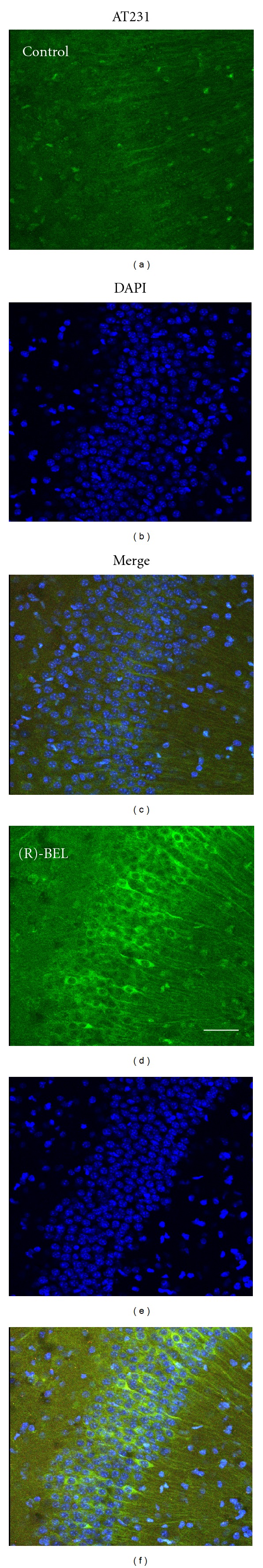
Inhibition of iPLA_2_ *γ* induces Tau phosphorylation in area CA1 of hippocampus. Cultured hippocampal slices from P301L Tau transgenic mice were treated with the iPLA_2_ *γ* inhibitor (R)-BEL. Slices were then processed for confocal immunofluorescence microscopy with an antibody recognizing Tau phosphorylation at Threonine 231 epitopes (AT231, in green) (AbCam, Cambridge, MA, USA. Dilution 1 : 750). When compared to controls (upper panel), immunostaining revealed increased phosphorylation in the CA1 region of cultured hippocampal slices incubated with 3 *μ*M (R)-BEL for a period of 12 h (lower panel). DAPI (in blue) was included in the mounting medium to label nuclei. This observation was qualitatively reproduced in hippocampal slices obtained in 3 different cultures. Scale bar = 25 *μ*m.

Normally, Tau predominantly localizes to neuronal axons where it modulates the stability and assembly of microtubules [128, 129]. In so doing, Tau generates a partially stable, but still dynamic, state in microtubules that is important for axonal growth and effective axonal transport [130]. In addition to binding microtubules, some but not all studies provide evidence that Tau can interact, either directly or indirectly, with actin and affect actin polymerization as well as the interaction of actin filaments with microtubules [131, 132]. Furthermore, Tau appears to interact with the plasma membrane and with several proteins involved in signal transduction [133–141]. From a pathological perspective, Tau dysfunction resulting from biochemical defects (i.e., aberrant phosphorylation, truncation, and glycosylation) has been proposed to be an important factor contributing to the initiation and development of several neuropathological conditions such as AD [142–147]. As discussed above, lower iPLA_2_ activity has been observed in AD brains and considering our hypothesis that iPLA2*γ* is an important factor controlling AMPA-mediated toxicity in the hippocampus, we anticipated that defect in iPLA_2_ *γ* activity can contribute to enhance Tau phosphorylation. Here, we are presenting the first experimental evidence that Tau become hyperphosphorylated after selective inhibition of iPLA_2_ *γ*. We first examined Tau phosphorylation levels at Ser199 residue following treatment of hippocampal slices with R-BEL and S-BEL, which preferentially block iPLA_2_ *γ* or iPLA2*β*, respectively (see chemical structures; Figure 1(a)). In initial experiments, we observed that hippocampal tissues were strongly and consistently stained with an antibody recognizing the phosphorylated Ser199 epitope of a Tau isoform of 62 kDa (Figure 1(b), top panels). As shown in Figure 1, staining for this hyperphosphorylated Tau isoform increased following iPLA_2_ *γ* inhibition by R-BEL. When the results were normalized with staining levels of Tau-5 (an antibody that recognizes Tau independent phosphorylation), it appears that R-BEL elevated levels of phosphorylated Tau at all concentrations tested, with a maximal increase of 120 ± 10% over control values in slices preincubated for 3 hr. However, the same analysis showed that phosphorylation of Tau at Ser199 was not altered by exposure to the iPLA_2_ *β* inhibitor S-BEL. It is noteworthy that levels of Tau-5 immunoreactivity in the hippocampal slices were not significantly changed by treatments with either R- or S-BEL, indicating that iPLA_2_ *γ* inhibition-induced increases in Ser199 phosphorylation do not depend on Tau synthesis and/or degradation. De-Paula and collaborators recently showed that injection of the dual cPLA_2_ and iPLA_2_ inhibitor methyl arachidonyl fluorophosphonate (MAFP) induced Tau phosphorylation at Ser214 [148]. In contrast to our results, however, they reported that Tau hyperphosphorylation was associated with a reduction in levels of total Tau [149], suggesting that inhibition of both cPLA_2_ and iPLA_2_ might influence several biochemical aspects of Tau proteins. Accordingly, recent experimental results have provided evidence that cPLA_2_ and iPLA_2_ activities can play divergent roles during spinal cord injuries [150]. We recently tested the effect of R-BEL-mediated iPLA_2_ *γ* inhibition on Tau subcellular localisation in CA1 pyramidal cells. Using organotypic hippocampal slice cultured from transgenic mice expressing human Tau, we observed that treatment with the specific iPLA_2_ *γ* inhibitor (R)-BEL for up to 12 h resulted in increases in Tau phosphorylation at the Thr231 site. High-resolution imaging showed that hyperphosphorylated Tau was primarily localized in the cell bodies and dendrites of hippocampal pyramidal neurons (see Figure 2).

One of the central hypotheses for AD pathogenesis is that the production of cytotoxic A*β* peptides impairs neuronal activity and leads to a decline in memory and cognition [151]. Some PLA_2_ enzymes may exacerbate A*β* cytotoxicity, as A*β* peptides stimulate cPLA_2_ *α* activity in neuronal cultures [86] and primary cortical astrocytes [152]; in addition, A*β*-induced learning and memory deficits in a transgenic mouse model of AD are prevented by genetic ablation of cPLA_2_ *α* activity in brain [152]. On the other hand, it has been well established that iPLA_2_ activity is essential for maintaining membrane phospholipid integrity by reducing peroxidative damage, especially that originating in mitochondria. In this regard, iPLA_2_ expression prevents the loss of mitochondrial membrane potential and attenuates the release of cytochrome c as well as of other apoptotic proteins, and ultimately reduces apoptosis in INS-1 cells exposed to staurosporine [153]. Furthermore, Kinsey et al. [95, 154] reported that a major component of PLA_2_ activity in mitochondria of rabbit renal proximal tubular cells is provided by iPLA_2_ *γ* and is of critical importance for the prevention of basal lipid peroxidation and maintenance of mitochondrial viability. Based on recent studies, it has been proposed that A*β*-induced neurotoxicity might derive from mitochondrial defects. Indeed, in vitro experiments have shown that A*β* peptides can be internalized by cells, imported into mitochondria and ultimately elicit mitochondrial dysfunctions [155]. Given its localization, it is thus tempting to propose that iPLA_2_ *γ* might represent an important cellular component that prevents mitochondrial dysfunctions. Experiments are required to determine whether iPLA_2_ *γ* overexpression activity might exert protective effects against A*β* peptide-induced mitochondrial dysfunctions.

From a pathological perspective, it has been demonstrated that iPLA_2_ activity is upregulated in the hippocampus of patients suffering from schizophrenia [156]. The precise implication of this iPLA_2_ dysfunction in the development of schizophrenia-related symptoms remains unknown. However, the results presented above would predict that upregulation of iPLA_2_ *γ* activity could eventually lead to reduction in GluR1-containing receptors. Interestingly, GluR1 downregulation has been reported to evoke striatal hyperdopaminergic activity [157], a well-established biological defect involved in schizophrenia-related symptoms. The potential relationship between iPLA_2_s and the dopaminergic system is reinforced by the fact that iPLA_2_ inhibition or knockdown in rat striatum, motor cortex and thalamus results in the apparition of Parkinson-related manifestations [158], which are also known to depend on dopamine dysfunction. Of course, future experiments will be required to establish the potential role of iPLA_2_ enzymes in stabilizing dopamine-mediated responses.

## 5. Conclusion

**Figure 3:**
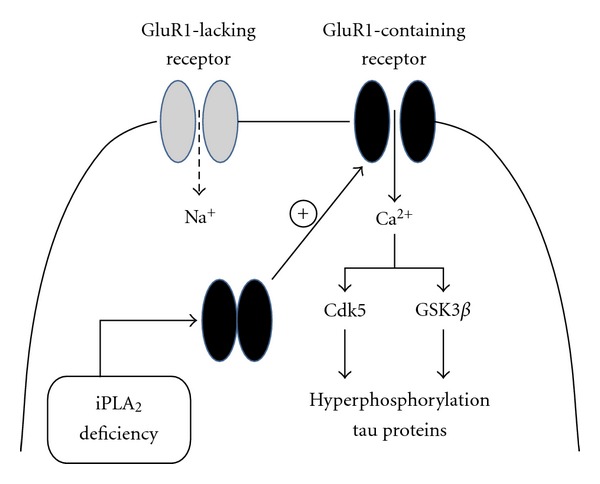
A putative model illustrating the potential implication of iPLA_2_ *γ* in Alzheimer's disease. In this simplified model, iPLA_2_ dysfunction leads to excessive delivery of GluR1-containing receptors to neuronal membranes. These receptors are more likely to be calcium-permeable and therefore to stimulate calcium influx and, eventually, Tau phosphorylation by calcium-dependent protein kinases such as Cdk5 and GSK-3*β*.

Here, we have summarized growing evidence linking iPLA_2_ *γ* activity to the stabilization of synaptic AMPA receptor properties in hippocampal neurons. First, it appears evident that without appropriate levels of iPLA_2_ *γ* activity in area CA1 of hippocampal slices synaptic stabilization of AMPA receptors, which is required for the expression of long-term changes in synaptic strength (i.e., LTP), is compromised. As mentioned previously, iPLA_2_ *γ* mRNAs and proteins are enriched with the endoplasmic reticulum (ER)-Golgi apparatus in several cell type, where they will be essential for diverse intracellular trafficking pathways, such as retrograde movement from the Golgi complex to the ER, transport of material from the trans-Golgi network to the plasma membrane, or recycling of membranes and receptors through endocytic pathways [21]. Since AMPA receptors trafficking from the ER-Golgi complex to postsynaptic membranes is critically involved in LTP [77], the iPLA_2_ *γ* isoform may be well suited to facilitate AMPA receptor translocation from intracellular pools to synaptic membranes during LTP. However, given their biochemical properties and localization, future experiments will be required to determine how the effects of iPLA2*γ* on LTP might derive from alterations of other cellular processes controlling synaptic stability such as regulation of arachidonic acid release, membrane fusion events, receptor trafficking pathways, and protein kinase activities. Besides, we also documented that iPLA2*γ* deficiency can destabilize synaptic GluR1 subunits of AMPA receptors in hippocampl membranes and accentuate glutamate-induced toxicity. In this line, iPLA2*γ*-null mice have been generated [159, 160] and were found to exhibit phenotypic abnormalities that include altered mitochondrial morphology, function, and lipid composition associated with hippocampal degeneration. Interestingly, we provided here preliminary evidence showing that iPLA_2_ *γ* activity appears to be important for stabilizing Tau phosphorylation in hippocampal pyramidal neurons, suggesting that downregulation of iPLA_2_ activity may contribute to the development of tauopathies in AD [161]. A putative biochemical model that could account for the potential influence of iPLA_2_ *γ* on Tau pathology is presented in Figure 3. Indeed, considering the growing evidence relating the importance of iPLA_2_ *γ* in physiological and pathological conditions, targeting iPLA_2_ *γ* activity may represent a potentially new therapeutic strategy to address several neurological disorders characterized with destabilisation of synaptic properties.

## Figures and Tables

**Table 1:** Calcium-independent group VI phospholipase A_2_ (iPLA_2_).

Group	Source	Molecular mass (kDa)	Feature	Alternate names
VIA-1	Human/Murine	84-85	8 ankyrin repeats	iPLA_2_
VIA-2	Human/Murine	88–90	7 ankyrin repeats	iPLA_2_ *β*
VIB	Human/Murine	88–91	Membrane-bound	iPLA_2_ *γ*
VIC	Human/Murine	146	Integral membrane protein	iPLA_2_ *δ*
VID	Human	53	Acylglycerol transacylase, triglycerol lipase	iPLA_2_ *ε*
VIE	Human	57	Acylglycerol transacylase, triglycerol lipase	iPLA_2_ *ξ*
VIF	Human	28	Acylglycerol transacylase, triglycerol lipase	PLA_2_ *η*
